# The Mechanism of Choline-Mediated Inhibition of Acetylcholine Release in Mouse Motor Synapses

**Published:** 2014

**Authors:** A. E. Gaydukov, P. O. Bogacheva, E. O. Tarasova, O. P. Balezina

**Affiliations:** Lomonosov Moscow State University, Faculty of Biology, Department of Human and Animal Physiology, Leninskie Gory, 1, build. 12, Moscow, 119234, Russia

**Keywords:** quantal content, ryanodine receptors, choline, α7-nicotinic acetylcholine receptors, SK channels

## Abstract

The mechanism of action of tonically applied choline, the agonist of α7 nicotinic acetylcholine receptors (nAChRs), to the spontaneous and evoked release of a neurotransmitter in mouse motor synapses in diaphragm neuromuscular preparations using intracellular microelectrode recordings of miniature endplate potentials (MEPPs) and evoked endplate potentials (EPPs) was studied. Exogenous choline was shown to exhibit a presynaptic inhibitory effect on the amplitude and quantal content of EPPs for the activity of neuromuscular junction evoked by single and rhythmic stimuli. This effect was inhibited either by antagonists of α7-nAChRs, such as methyllycaconitine and α-cobratoxin, or by blocking SK-type calcium-activated potassium (KCa) channels with apamin or blocking intraterminal ryanodine receptors with ryanodine. A hypothesis was put forward that choline in mouse motoneuron nerve terminals can activate presynaptic α7-nAChRs, followed by the release of the stored calcium through ryanodine receptors and activation of SK-type KCa channels, resulting in sustained decay of the quantal content of the evoked neurotransmitter release.

## INTRODUCTION

Although postsynaptic nAChRs in the motor synapses of the skeletal muscles of vertebrates have been thoroughly studied [1-3], data on presynaptic ones is rather scarce and contradictory. Immunohistochemical and pharmacologic tests demonstrate that there are several types of presynaptic nAChRs in motor synapses [4-7]. At the same time, the location and functions of the specific nAChRs remain poorly studied, especially those of α7-nAChRs [8, 9] that are characterized by a comparatively high calcium-ion conductivity [10-12]. In contrast to the central nervous system where activation of presynaptic α7-nAChRs with ACh or selective agonists (choline, nicotine) typically facilitates neurotransmitter release [13-16], inhibition of the release in peripheral motor synapses has been reported [5, 17]. In our previous research, activation of α7-nAChRs with small doses of nicotine triggered calcium-dependent inhibition of the evoked release of acetylcholine in rhythmically stimulated neuromuscular junctions of mouse, which could be prevented by using methyllycaconitine, a selective antagonist of α7-nAChRs [18]. The mechanisms of this inhibition remain unclear. Due to this fact, presynaptic α7-nAChRs in the present work were activated by their selective agonist choline in order to assess its ability to suppress the evoked ACh release and to study the mechanisms of this effect.

## EXPERIMENTAL

**Object of research**

Experiments were carried out using isolated neuromuscular preparations of the diaphragm *(m. diaphragma – n. phrenicus) *of mature (30) male mice of the 129/Sv line provided by the Anokhin Institute of Normal Physiology of the Russian Academy of Sciences (Moscow, Russia). A total of 27 animals were used. The mice were managed in accordance with the Directive 86/609/EEC regulating the use of laboratory animals. The procedure was approved by the Bioethics Commission of the Department of Biology of the Moscow State University. The mice were euthanized by quick decapitation.

**Electrophysiology**

The dissection of muscle fiber allowing one to simultaneously record both a spontaneous and non-reduced evoked release of the neurotransmitter was performed according to the standard protocol [5, 17, 18]. The left half of the diaphragm with the phrenic nerve was put into a 3-mL camera and rinsed with an oxygenated (95% O2, 5% CO_2_) Liley buffer (pH 7.2–7.4, 135 mM NaCl, 4 mM KCl, 0.9 mM NaH_2_PO_4_, 2 mM CaCl_2_, 1 mM MgCl2, 16.3 mM NaHCO_3_, 11 mM glucose) at room temperature. All experiments were carried out at 20–22 °C. MEPPs and EPPs were recorded using intracellular glass microelectrodes filled with 2.5 M KCl (resistance at the microelectrode tip was 15–20 MΩ). Single EPPs were detected upon stimulation of the phrenic nerve with suprathreshold impulses of 0.3 Hz frequency (at least 30 stimuli). When studying the rhythmic synaptic activity, the phrenic nerve was stimulated with short trains of stimuli (50 stimuli 0.1 ms long each, frequency of 50 Hz). Signals were registered by an Axoclamp-2B amplifier (Molecular Devices) and recorded using an L-Card E-154 analog-to-digital converter (with PowerGraph interface) into the PC hard drive. The data were processed using the MiniAnalysis software (Synaptosoft). Controls included MEPP and EPP recordings from 5 or more different synapses under normal conditions and after the substances under study had been administered in a certain order. The synaptic activity was registered during 1–1.5 h. At least 3 neuromuscular preparations were used in each series of experiments.

**Substances and solutions**

Choline, methyllycaconitine, apamin (Sigma, USA), and ryanodine (Enzo Life Sciences, USA) were used. A high-affinity blocker of α7-nAChRs, namely the long-chain α-cobratoxin (extracted from *Naja naja kaouthia*) [19-21], was kindly provided by Yu.N. U tkin, the head of the Laboratory of Molecular Toxicology of the Shemyakin–Ovchinnikov Institute of Bioorganic Chemistry, Russian Academy of Sciences (Moscow, Russia).

**Data analysis and statistics**

We estimated the amplitude, variation of MEPPs and EPPs with time, the MEPP frequency, and the quantal content of EPP (the latter was calculated as the ratio between the mean EPP amplitude corrected for non-linear summation [22] to the mean MEPP amplitude). The statistical significance of the difference between sample groups was assessed using the Student’s t-test and Mann–Whitney test. The significance level of the differences between two sample groups was 0.05 (*n *– being the number of synapses studied).

## RESULTS

**Fig. 1:**
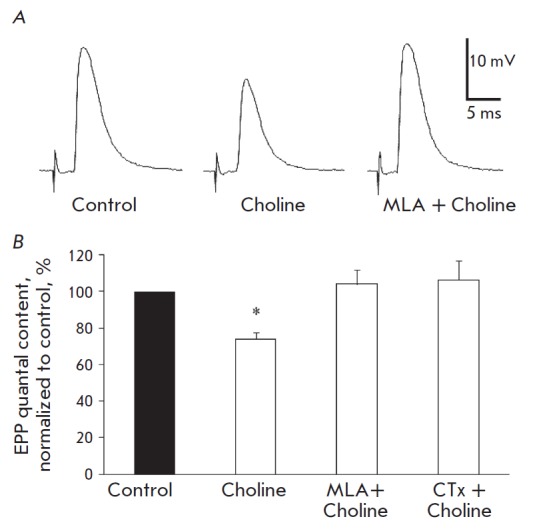
Inhibitory effect of exogenous choline on the single (0.3 Hz) evoked release of neurotransmitter mediated by its influence on α7-nAChRs. *A *– averaged recordings of single EPPs in controls, in the presence of choline (100 uM) and in the presence of both choline (100 uM) and MLA (20 nM). *B *– quantal content of single EPPs in controls, in the presence of choline and in the presence of choline and previously administered MLA and CTx (5 nM). The Y axis shows the quantal content of EPPs (% compared to the control), *p  < 0.05 compared to the controls

In the first series of experiments, the muscle was rinsed with a 100-μM choline solution for 40 min. The characteristics of MEPPs and the single-evoked EPPs were analyzed. No statistically significant changes in the membrane potential (MP) in the postsynaptic membrane were revealed during choline perfusion ((the average MP in the controls was –39.16 ± 1.13 mV (n = 18) and –40.06 ± 1.18 (n = 19) in the presence of choline). Choline reduced the EPP amplitude by over 25% on average as compared to the control *(Fig. 1A). *The effect developed within 10–15 min after the administration of choline and remained unchanged during the next 30 min. The changes in amplitude, temporal characteristics, and MEPP frequency were not statistically significant; the decline in the EPP amplitude was caused by a decrease in the quantal content of EPPs from 34.20 ± 2.56 in the control to 25 ± 2.56 in the presence of choline (p < 0.05) *(Fig. 1B). *In additional experiments on intact (non-dissected) neuromuscular preparations, 100-μM choline caused no significant changes in the MEPP amplitude (1.49 ± 0.07 mV in controls (n = 17) and 1.52 ± 0.11 (n = 17, *p * < 0.05) in the presence of choline). Compared with the controls, the MEPP frequency and its variation with time in the presence of choline were not significantly different. Thus, the decline in the quantal content of EPPs in the presence of choline at the same MP and MEPP values indicates that choline has a presynaptic inhibitory effect on the evoked quantal release of ACh.

Choline-induced inhibition of the quantal content of the single EPP could be prevented by a selective blocker of α7-nAChRs, methyllycaconitine (MLA), administered in a concentration of 20 nM. When administered for 15–30 min, MLA caused no statistically significant changes in MEPPs and EPPs; however, the amplitude and quantal content of single EPPs did not decrease in the presence of methyllycaconitine choline, either (*Fig. *1A,B). A similar result—the prevention of the inhibitory effect of exogenous choline on the quantal content of single EPPs—was obtained with the aid of another selective blocker of α7-nAChRs, the long-chain α-cobratoxin (CTx) administered in a concentration of 5 nM *(Fig. 1B). *This means that choline, when administered tonically, facilitates the inhibition of the evoked release of ACh precisely by activating α7-nAChRs.

**Fig. 2:**
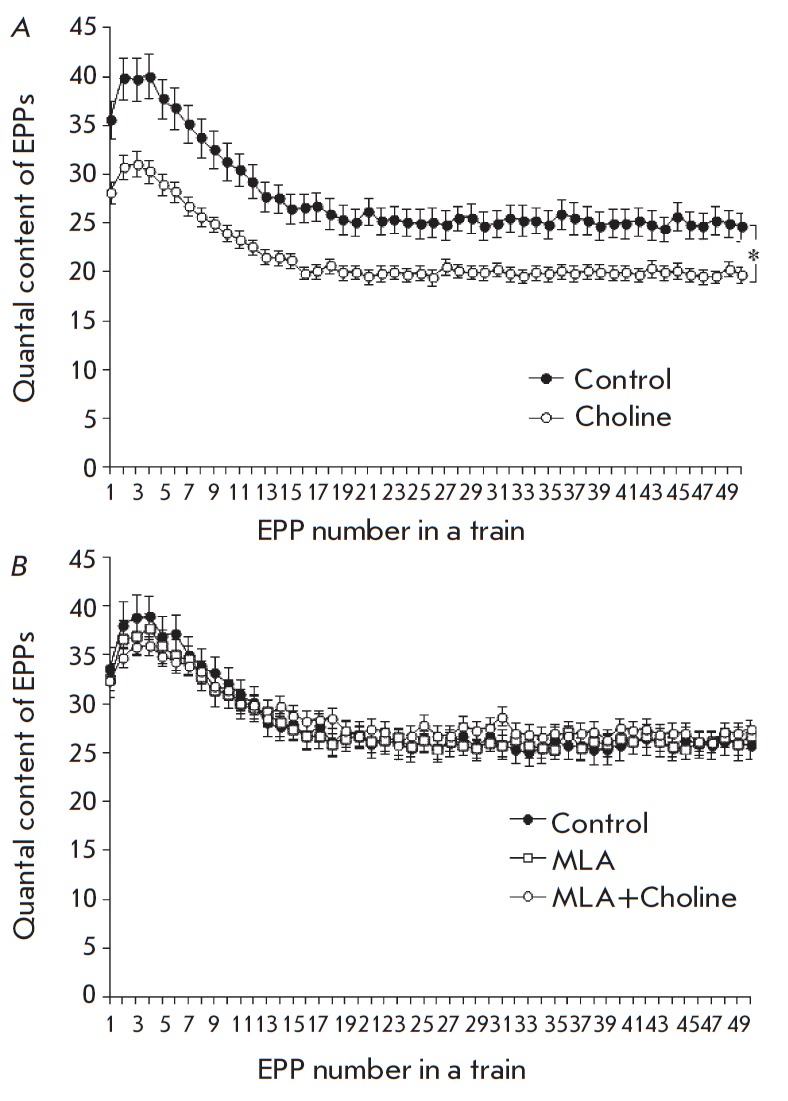
Change in the quantal content of EPPs during the short train of stimuli at a rhythmical activity of synapses of 50 Hz. *A *– in controls and upon administration of 100 •uM choline. *B *–in controls, in the presence of 20 nM MLA and upon administration of choline subsequent to MLA. The Y axis shows the quantal content of EPP; the X axis shows the number of EPPs in the train, *p < 0.05 compared to controls

The following series of experiments was aimed at investigating choline-induced responses to short trains consisting of 50 EPPs (50 Hz, 1s). Administration of 100-μM choline for 40 min showed the same decrease in the amplitude and quantal content as that observed for single-evoked EPPs; this effect was recorded for the first and all following EPPs in the train. It developed during the first 10–15 min of choline administration and remained unchanged for the next 30 min. There, the EPP train pattern did not change: identically to the controls, we observed short-term facilitation of the synaptic transmission in the beginning of the train, which was followed by the depression continuing into a lower stable level of EPPs compared to the first one (a plateau) *(Fig. 2A). *Preliminary administration of selective blockers of α7-nAChRs, 20nM MLA (*Fig. 2B*) or 5 nM CTx, to the neuromuscular preparation changed neither the MEPP parameters nor quantal content of EPP of the high-frequency short train. Meanwhile, choline administered alongside the mentioned blockers of α7-nAChRs had no inhibitory effect on the quantal content of EPP in trains* (Fig. 2B).*

**Fig. 3:**
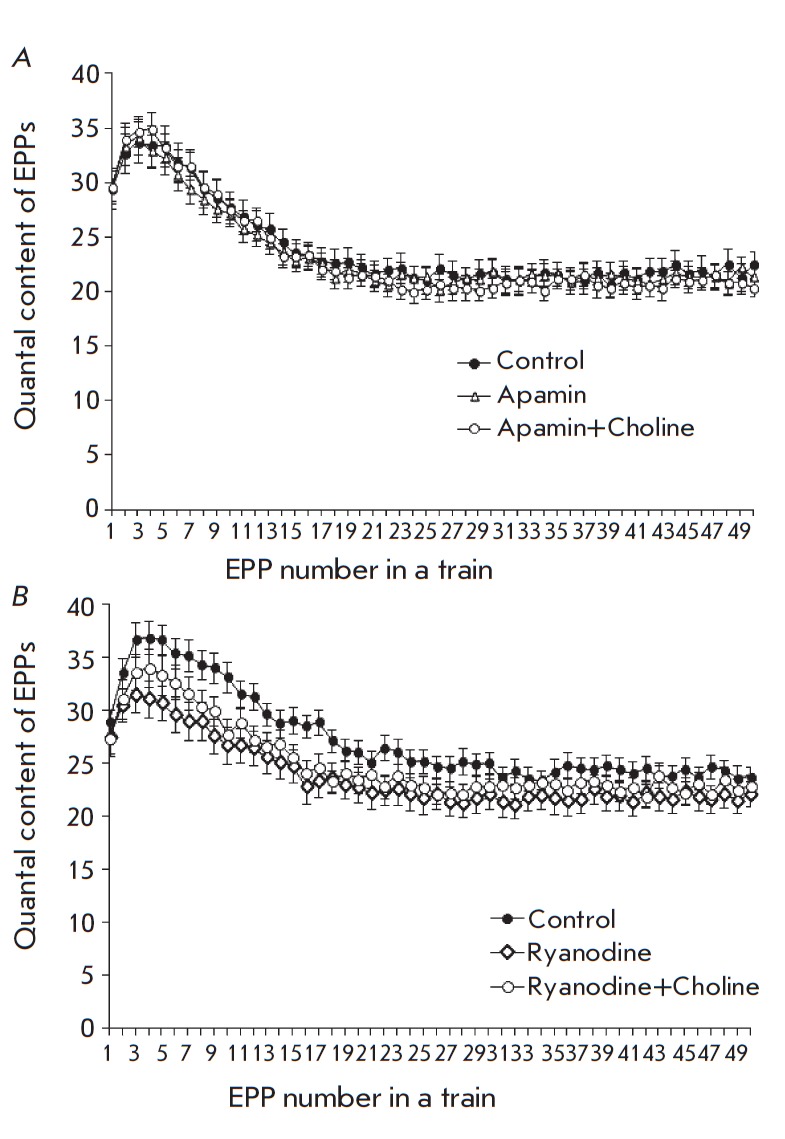
Change in the quantal content of EPPs during the short train of stimuli at a frequency of 50 Hz. *A *– in controls, in the presence of 200 nM apamin, and in the presence of both 100 uM choline and apamin. *B *– in controls, in the presence of 3 uM ryanodine, and in the presence of both 100 uM choline and ryanodine. The Y axis shows the quantal content of EPPs; the X axis shows the number of EPPs in the train

To elucidate the mechanism of the inhibitory effect of choline, an assumption was made that choline-induced activation of α7-nAChRs letting calcium in the terminal generates a calcium signal that can activate SK K_Ca_ channels similar to the pathway of the inhibitory effect of ACh on the impulse activity of some other excitable cells [23, 24]. A selective blocker of SK K_Ca_ channels, apamin, was administered to the muscle in a concentration of 200 nM to verify this hypothesis. Apamin alone provided no statistically significant changes in the amplitude and quantal content of the single or rhythmically generated EPPs, but 100-μM choline administered along with it lost its ability to inhibit the quantal content of EPPs in trains *(Fig. 3A). *All these facts allowed us to assume that the inhibitory effect of exogenous choline depends on calcium and is based on the choline-induced activation of the calcium influx into the terminal via channels of α7-nAChRs, which activates potassium SK-channels and the outgoing potassium current. The ensuing membrane hyperpolarization suppresses the voltage-dependent calcium channels in active zones, thus diminishing the possibility of the evoked ACh release.

According to publications, SK channels can be activated by calcium from different sources [25]. Thus, for instance, the activity of SK channels in certain hippocampal synapses [24] rises due to the calcium-triggered release of calcium from stores caused by the influx of calcium from the outside through the channels of α7-nAChRs. That is why the next series of experiments were aimed at elucidating the possible involvement of ryanodine receptors and the release of calcium from the calcium stores of motor terminals in the mechanisms of the calcium-dependent inhibitory effects of choline employing SK potassium channels.

Application of ryanodine in a concentration that reciprocally blocks ryanodine receptors (3 μM) to the muscle showed no statistically significant changes in the amplitude and quantal content of EPPs but insignificantly worsened the transmission in the beginning of the short train of EPPs (*Fig. 3B). *With a ryanodine presence (3 μM), the subsequent application of choline did not decrease the amplitude or quantal content of EPPs in the train (*Fig. 3B). *This fact demonstrates that calcium-dependent choline-induced inhibition of the evoked release of ACh requires not only α7-nAChRs, but also the release of calcium from stores.

## DISCUSSION

The effects discovered by administering exogenous choline (100 μM) and selective blockers of α7-nAChRs (methyllycaconitine and a-CTx), along with the impact of an inhibitor of SK channels (apamin) and that of the blocker of ryanodine receptors (ryanodine), elucidated the mechanism of the inhibitory effect of choline on the evoked ACh release.

The ability of certain endogenous and exogenous agonists of neuronal nAChRs when applied briefly (several seconds) and in high (millimolar) concentrations to inhibit ACh release in motor synapses has been reported earlier in a number of studies [5, 8, 17]. However, those studies specified neither the type of presynaptic nAChRs mediating these effects nor the mechanism of the latter. Choline is known to be a full selective agonist of α7-nAChRs and at the same time an activator of the M1-choline receptors located on the terminals and motor synapses of Schwann cells [26]. However, the publications state that choline activates these receptors when administered in doses that are considerably higher than those used in our study [27, 28]. Apart from that, the selective activation of the M1-choline receptors of motor synapses facilitates the release of neurotransmitter [29, 30] and, thus, cannot be a reason for the discovered inhibitory effect of exogenous choline on ACh release. That is why in our attempts to explain the discovered choline effects we relied on the well-documented and widely known facts of choline ability to selectively activate the α7-nAChRs of nerve terminals [31, 32].

According to the protocol used, choline was applied tonically (during several dozens of minutes) at a low concentration of 100 μM, which does not reach EC_50_ for activating α7-nAChRs (0.5–1.5 mM) [31, 33]. It is commonly known that α7-nAChRs belong to the family of rapidly desensitizing choline receptors [34]. However, according to the desensitization model of α7-nAChRs, low (not exceeding EC_50_) concentrations of agonists lead to prolonged opening of the channel of α7-nAChRs with insignificant desensitization or blockage of the open channel at negative (hyperpolarized) MP values [32]. The fact that choline-induced decay of the quantal content of EPPs can be prevented by blockers of α7-nAChRs means that the effect of choline in this particular concentration (100 μM) is mediated by the activation, not desensitization, of neuronal nAChRs on the presynaptic membrane. The prolonged effects of choline might be due to the processes taking place upon activation of α7-nAChRs. It has recently been shown on preterminal axons of hippocampal neurons that even short-term activation (10 min) of nAChRs with exogenous agonists may lead (after the immediate effects) to a long-term (30 min and more) intracellular rise in the calcium content, activation of CaMKII and other enzymes, accompanied by a long-term increase of the neurotransmitter release [35].

In our study of peripheral synapses, attempts to activate presynaptic α7-nAChRs with choline revealed another effect, namely the long-term inhibition of the neurotransmitter release caused by the involvement of SK K_Ca_ channels. These channels have been described for motoneuron nerve terminals in rodents [36]. It also has been shown that they might be involved in the regulation of the spontaneous MEPP frequency [37]. Our work is the first to report the activation of SK channels and their involvement in the possibly mediation of the inhibitory impact of choline on the evoked ACh release. Similar examples of the response of SK channels to the activation of α7-nAChRs have been described for the central synapses of hair cells [23] and hippocampal neurons [24].

Administering ryanodine as a blocker of ryanodine receptors demonstrated another necessary component that mediates the inhibitory effects of choline — ryanodine- dependent release of calcium from stores. In the central nervous system, functional coupling of α7-nAChRs to ryanodine receptors strengthens the calcium signal in terminals and facilitates the release of ACh and other neurotransmitters [14, 38, 39]. We were first to demonstrate that in peripheral synapses, on the contrary, functional interaction between α7-nAChRs and the ryanodine receptors of calcium stores decreases the evoked neurotransmitter release due to the activation of SK K_Ca_ channels. α7-nAChRs are apparently located in the terminals of motoneurons, far from the exocytosis sites, but spatially close to certain perimembrane cisterns of ryanodine calcium stores; thus, the entire complex can activate SK potassium channels. A similar interaction between α7-nAChRs, ryanodine receptors, and SK channels was described for hippocampal interneurons at the postsynaptic level [24] and in hair cells [40]. In both cases, it slowed down the neuronal activity.

It is widely known that spatial diffusion of the combined action of extracellular ACh and its derivate, choline, in the central nervous system may regulate the activity of the extrasynaptic and perisynaptic α7-nAChRs located on preterminal axons, neuronal dendrites, and bodies of glial cells [41]. For peripheral axons and the terminals of motoneurons, a regulation that would employ ACh and choline has not been reported yet. In neuromuscular junctions, the rate of ACh release and the level of AChE activity are significantly higher compared to those in the central cholinergic synapses [41]. Therefore, the prolonged activity of synapses and ACh hydrolysis must significantly increase the level of endogenous choline in the synaptic cleft. Its diffusion from the cleft and the activation of presynaptic α7-nAChRs might serve as a negative feedback mechanism of endogenous auto-regulation of ACh release. Nevertheless, we were not successful in establishing a response by endogenous choline to the ACh release upon single and short-train stimulation of synapses. Contrary to expectations, administration of blockers of α7-nAChRs failed to cause any changes in the quantal content of the single EPPs and short trains of EPPs(50 EPP, 50 Hz). A longer and more intensive action of motor synapses is probably required to accumulate endogenous choline. The same relates to its diffusion (spillover) from the cleft and development of an inhibitory effect, especially when presynaptic α7-nAChRs are distanced from the exocytosis sites (e.g., preterminal α7-nAChRs in central synapses) [42]. This concept was confirmed by the results of experiments on the rat diaphragm, where the ability of blockers of α7-nAChRs to prevent a decline in the quantal content of EPPs could be detected only on condition that it was evolving during a prolonged (several hours) low-frequency activity of synapses [17].

## CONCLUSIONS

Our study has demonstrated the tonic effect of choline administered in concentrations relatively low on the activation of α7-nAChRs to cause long-term inhibition of the ACh release.

We were the first to reveal the mechanism of this inhibition. It consists in the activation of presynaptic axonal α7-nAChRs with choline, the subsequent release of calcium from stores through ryanodine receptors, and activation of SK KC_a_ channels in mouse motor terminals. We cannot rule out other possible participants in this mechanism; such as certain calcium-dependent enzymes. However, further research is required to elucidate this point. It is also interesting to test whether choline-dependent inhibition of the neurotransmitter release can contribute to the fatigue of neuromuscular transmission at a prolonged intensive work of motor synapses in mammals.

## Abbreviations

ACh: acetylcholine
MEPP: miniature endplate potential
nAChRs: nicotinic acetylcholine receptors
EPP: endplate potential
